# Recent Advances in the Seed-Directed Synthesis of Zeolites without Addition of Organic Templates

**DOI:** 10.3390/nano12162873

**Published:** 2022-08-21

**Authors:** Shujie Dai, Yichang Yang, Jinghuai Yang, Shichang Chen, Longfeng Zhu

**Affiliations:** 1 Key Laboratory of Advanced Textile Materials and Manufacturing Technology, Ministry of Education, Zhejiang Sci-Tech University, Hangzhou 310018, China; 2 College of Biological, Chemical Sciences and Engineering, Jiaxing University, Jiaxing 314001, China; 3 College of Chemical Engineering, Zhejiang University of Technology, Hangzhou 310014, China

**Keywords:** zeolite, green synthesis, seed-directed, organotemplate-free, utility

## Abstract

Zeolites have been widely employed in fields of petroleum refining, fine chemicals and environmental protection, but their syntheses are always performed in the presence of organic templates, which have many drawbacks such as high cost and polluted wastes. In recent years, the seed-directed synthesis of zeolites has been paid much attention due to its low-cost and environmentally friendly features. In this review, the seed-directed synthesis of Al-rich zeolites with homonuclear and heteronuclear features, the seed-directed synthesis of Si-rich zeolites assisted with ethanol and the utility of seed-directed synthesis have been summarized. This review could help zeolite researchers understand the recent progress of seed-directed synthesis.

## 1. Introduction

With the continuous development of the economy, human beings have an increasing demand for energy. At present, fossil fuels as the main energy still play an important role, but it results in environmental problems that cannot be ignored. Thus, how to use energy more efficiently is an important problem. Now, it has been proved that catalysis is one of the main ways for the efficient use of energy. In the research of catalysis, the catalyst is the core factor of catalytic technology and the development of catalytic materials would promote the development of the catalyst and catalytic process. Among many catalytic materials, zeolites have been widely used in industrial fields such as adsorption, separation and catalysis because of their uniform pore structure, high surface area and excellent stability [1,2,3,4]. Therefore, the researchers are devoted to the designed synthesis of zeolites, such as the generation of novel zeolite structures [5,6,7,8,9,10,11,12,13], the development of a novel route for synthesizing zeolites [14,15,16,17,18,19], the control of zeolite morphologies [20,21,22,23,24], the preparation of micro/mesoporous zeolites [25,26,27,28,29] and the synthesis of zeolites from natural aluminosilicates (e.g., montmorillonite, kaolin, halloysite) as the silica/alumina green sources [30,31,32,33,34,35].

Notably, the modern synthesis of zeolites often requires the employment of organic templates. Due to the structural diversity of organic templates, a variety of new zeolite structures have been successfully synthesized. However, the employment of organic templates also has many shortcomings, as follows: (1) most of the organic templates are expensive and toxic, which greatly increases the synthesis cost; (2) to obtain opened micropores, it would consume the energy for the removal of organic templates during the process of calcination at a high temperature as well as result in generating a large amount of harmful gases such as NO_x_ and CO_2_; (3) the zeolite frameworks are easily damaged during high temperature calcination [16]. Obviously, the use of organic templates limits the further application of zeolites from the perspective of both the elimination of environmental pollution and the utilization of energy. Thus, it is highly desirable to synthesize zeolites in the absence of organic templates. At present, three methods for synthesizing zeolites under organotemplate-free conditions have been reported, including adjusting starting gels, the introduction of seed solution in the gels and addition of solid zeolite seeds (named as seed-directed synthesis). In particular, the seed-directed synthesis of zeolites has been a hot topic and a series of Al-rich zeolites have been synthesized in the presence of homonuclear and heteronuclear seeds, such as *BEA, MTT, CHA, KFI, etc. In addition, a variety of all (high) silica zeolites can be successfully synthesized by the combined strategy of both seed directing and ethanol filling, such as MFI, MTT, *MRE, SOD, etc. On this basis, many zeolites with special properties can also be obtained by using seed-directed synthesis. Herein, we briefly summarize the recent advances in the seed-directed synthesis of zeolites without the addition of organic templates, including the seed-directed synthesis of Al-rich zeolites with homonuclear and heteronuclear features, the seed-directed synthesis of Si-rich zeolites assisted with ethanol and the utility of seed-directed synthesis.

## 2. Seed-Directed Synthesis of Al-Rich Zeolites

The seed-directed route opens a new door for synthesizing zeolites with Al-rich features, which are being simple, low-cost and environmentally friendly. In addition, it also has many advantages including accelerating the crystallization rate, increasing the degree of crystallinity and avoiding the generation of impurity phases and controlling the crystal size. The seed-directed synthesis of zeolites is divided into the homonuclear and heteronuclear growth. The details of the seed-directed synthesis of Al-rich zeolites have been shown in Table 1.

### 2.1. Homonuclear Growth

Zeolite products have the same structures with the targeted zeolite seed added in the starting gel, which is called a homonuclear growth of seed-directed synthesis. Currently, it has been reported that more than 20 zeolites could be successfully synthesized using this route, such as *BEA [36,37,38], MTT [39], CHA [40,41,42], MWW [43,44], SZR [45], MSE [46], KFI [47,48] and so on. Considering the space limitation of this review, it would mainly introduce the zeolites that have been widely used in the industry including *BEA, MTT, CHA and MWW. 

#### 2.1.1. *BEA

Beta zeolite has a three-dimensional structure with a 12-membered ring, which shows excellent performance in the fields of petroleum refining and fine chemicals due to its good thermal stability, unique hydrophobicity and excellent catalytic performance [76]. The conventional synthesis of Beta zeolite is usually directed by tetraethylammonium hydroxide (TEAOH) as an organic template. As a result, to obtain the opened micropores, the organic template needs to be removed by high-temperature calcination, which not only causes great economic loss and energy consumption, but also releases a large amount of greenhouse gases and harmful gases when the organic template is decomposed. Therefore, the organotemplate-free synthesis of Beta zeolite is highly desirable.

**Figure 1:**
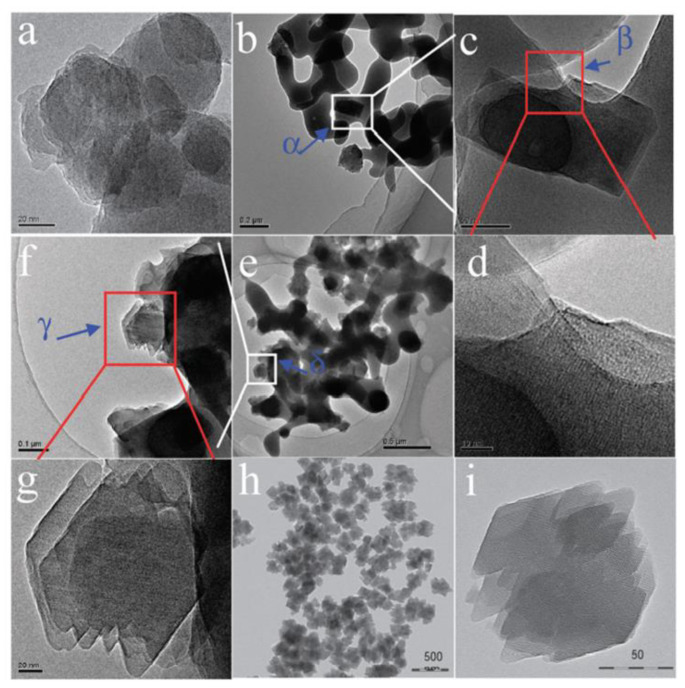
TEM images of Beta-OTF products crystallized for (**a**) 1, (**b**–**d**) 4, (**e**–**g**) 8 and (**h**,**i**) 18.5 h; areas of α, β, γ and δ in the Figure (**b**,**c**,**e**,**f**) are enlarged as Figure (**c**,**d**,**f**,**g**). Reprinted with permission from ref. [37]. Copyright 2011, The Royal Society of Chemistry.

Xie et al. [36] reported for the first time that Beta zeolite with high crystallinity (named as Beta-OTF) could be successfully obtained by adding about 10% calcined Beta zeolite as the seed into the starting gel at 140 °C for 18.5 h in the absence of any organic template. The N_2_ adsorption curve of the Beta-OTF demonstrated that the zeolite already had opened micropores, thus completely avoiding the calcination process and ensuring high crystallinity. In addition, the crystallization mechanism of Beta zeolite synthesized from the seed-directed route was studied by transmission electron microscopy (TEM), X-ray diffraction (XRD) and scanning electron microscopy (SEM), which displayed that the growth process of Beta-OTF followed the “core-shell” mechanism, as shown in Figure 1. In particular, the yield of Beta-OTF from seed-directed synthesis is low (about 30–40%) [37]. Subsequently, Yokoi et al. [38] displayed that an Al-rich suspension precursor was used for synthesizing Beta zeolite, which could greatly increase the product yield (about 80%). Notably, Beta zeolite synthesized from a seed-directed route is extremely Al-rich (the Si/Al ratio is only about 4), which is much lower than that of Beta zeolite synthesized by using an organic template (Si/Al ratio over 12) [77,78,79]. In addition, the Al-rich Beta zeolite has very stable four-coordinated Al species and good thermal and hydrothermal stability, which provide a new path for the development of high-efficiency zeolite catalysts with a highly acidic density in the future.

#### 2.1.2. MTT

ZSM-23 zeolite with a one-dimensional 10-membered ring [80] exhibits an excellent performance in a series of catalytic reactions, such as methanol to hydrocarbon [81,82,83] and isomerization [39,84]. Unfortunately, organic templates were necessary in its synthesis, such as isopropylamine [85], pyrrolidine [86], N,N-dimethylformamide [86], diquat-7 [87], etc.

To avoid the use of the above organic templates, Wu et al. [39] developed a method for synthesizing ZSM-23 zeolite by a seed-directed route (designated as ZJM-6). They studied, in detail, the effects of various factors such as the amount of seeds, the Si/Al ratio and H_2_O/SiO_2_ on the synthesis of ZSM-23 zeolite. The experimental results showed that the Si/Al ratio of ZJM-6 zeolite was about 20, which was far lower than that of the conventional ZSM-23 zeolite synthesized with pyrrolidine as an organic template (the Si/Al ratio was about 32–62). It is noteworthy that the crystallization of ZJM-6 takes only 5 h to obtain a targeted product with high crystallinity, which greatly shortens the crystallization time. Furthermore, due to the presence of more acidic sites in Al-rich ZJM-6 than conventional ZSM-23, the conversion of ZJM-6 (10.4%) was higher than that (4.1–9.4%) of conventional ZSM-23 zeolite in the catalytic isomerization of *m*-xylene to *p*-xylene. Therefore, it confirmed that the Al-rich feature of the ZSM-23 zeolite synthesized from the seed-directed route enhanced the catalytic activity of the reaction. Very interestingly, the as-synthesized ZJM-6 zeolite product could be used as zeolite seeds again to prepare ZJM-6-2 zeolite in the next run, which provides a green method for synthesizing ZSM-23 zeolite that completely avoids the use of organic templates.

#### 2.1.3. CHA

CHA zeolite has a three-dimensional eight-membered ring pore structure [88]. SSZ-13 zeolite, as a typical representative one, has been widely used in methanol-to-olefin (MTO) [89,90] and the selective catalytic reduction of NO with ammonia (NH_3_-SCR) [91,92]. In 1985, Zones et al. [93] firstly synthesized SSZ-13 zeolite using an expensive and toxic organic template of N,N,N-trimethyl-1-adamantammonium hydroxide (TMAdaOH) under hydrothermal conditions, which limited its further application. Subsequently, Martín et al. [94] synthesized SSZ-13 zeolite using the cheap tetraethylammonium hydroxide (TEAOH) as an organic template, which greatly reduced the preparation cost. However, the use of an organic template is not environmentally friendly. Therefore, it is highly desirable to synthesize SSZ-13 zeolite in the absence of any organic templates.

**Figure 2:**
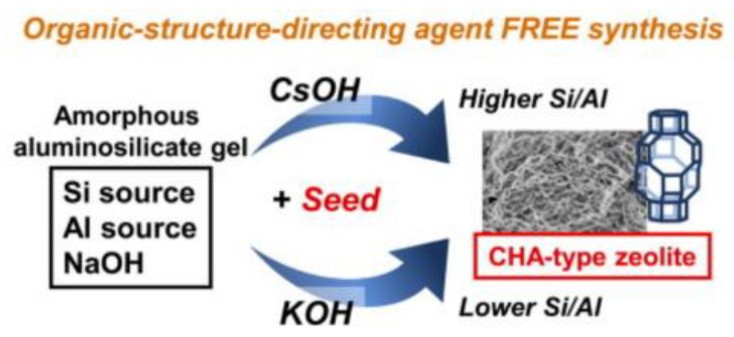
Schematic diagram of the CHA zeolite by a seed-assisted method in the absence of organic structure-directing agents. Reprinted with permission from ref. [40]. Copyright 2014, Elsevier.

In 2014, Takashi et al. [40] firstly synthesized SSZ-13 zeolite with an Si/Al ratio of 4 to 5 by a seed-directed route under an organotemplate-free condition. The key to successful synthesis was to add the zeolite seeds and introduce K^+^ or Cs^+^ into the synthetic system, as shown in Figure 2. At the same time, it was found that the replacement of K^+^ with Cs^+^ could increase the Si/Al ratio of SSZ-13 zeolite and the addition of B^3+^ in the initial gel could increase the Si/Al ratio of the product as high as 6.1.

In 2018, Wang et al. [41] successfully synthesized the micro-spherical hierarchical porous SSZ-13 zeolite with abundant mesopores via a seed-directed route using aluminum isopropoxide as an aluminum source. In the MTO reaction, the SSZ-13 zeolite showed the methanol conversion was 100% and the ethylene and propylene selectivity were over 90% plus the longer lifetime than that of the conventional SSZ-13 zeolite. The seed-directed synthesis of SSZ-13 zeolite not only significantly reduced the production cost and environmental pollution, but also shortened the crystallization time. However, the crystal size of SSZ-13 zeolite obtained by this method was 3–6 μm.

However, the micro-scale crystal size of the SSZ-13 zeolite affects their performance in gas adsorption [79] and catalysis [95] due to its diffusion limitations of the micropore. In 2020, Debost et al. [42] reported the direct synthesis of nanoscale SSZ-13 zeolite in a colloidal suspension containing a mixture of inorganic cations (Na^+^, K^+^, and Cs^+^) in the absence of an organic template. As a result, the obtained product showed the crystal size was approximately only 42 nm (thickness, along the c-axis) × 189 nm (width, in the ab-plane). The nanosized SSZ-13 zeolite improved its pore accessibility and thus exhibited an excellent CO_2_ adsorption capacity.

#### 2.1.4. MWW

MWW zeolite owns the two-dimensional sinusoidal 10-membered ring network channels (0.49 nm × 0.59 nm) in the inner layer and 12-membered ring super-cages between the layers (0.71 nm × 1.82 nm) [96], mainly including MCM-22, MCM-49, SSZ-25, MCM-36, ITQ-2, MCM-56, etc. Due to its unique pore structure and acidic characteristics, it exhibits an excellent performance in the alkylation of benzene with ethylene and propylene [97], the dehydrogenation skeletal isomerization of *n*-butane [98] and the aromatization of *n*-butane [99]. The conventional synthesis of MWW zeolites is not sustainable by using toxic and costly hexamethyleneimine (HMI) as an organic template [100].

Kamimura et al. [43] reported the first seed-directed synthesis of MWW zeolite. In this synthesis, the uncalcined MCM-22 zeolite was synthesized by the conventional method used as with zeolite seeds, which was added into a sodium-aluminosilicate gel system without the addition of an organic template preheated at 120 °C for 5 h in advance. The MCM-22 zeolite with high crystallinity can be obtained under static condition at 160 °C for 4 d. In the crystallization process, before the spontaneous nucleation of MOR zeolite, the more MWW zeolite seeds that were added, the larger the growth surface area provided could be, thereby obtaining MCM-22 zeolite with high quality. More recently, Xie et al. [44] used ultrasonic aging technology for the first time to synthesize MCM-49 zeolite by a seed-assisted route without the addition of an organic template. During the crystallization process, the ultrasonic treatment not only maintained the integrity of the zeolite seeds, but also promoted the entry of the aluminosilicate species into the solid phase and thus facilitated the uniformity of the system, which was helpful for the crystallization of MCM-49 zeolite. This above study develops a novel route for the efficient, eco-friendly and facile seed-directed synthesis of MCM-49 zeolite.

### 2.2. Heteronuclear Growth

Zeolite products have different structures but partly the same composite building units with the targeted zeolite seeds added in the starting gel, which is called the heteronuclear growth of seed-directed synthesis. In recent years, the synthesis of FER [73] and NES [75] has been reported typically.

#### 2.2.1. FER

FER zeolite has a two-dimensional pore structure in which an 8-membered ring (0.48 × 0.35 nm) and 10-membered ring (0.54 nm × 0.42 nm) are interlaced with each other [79]. Due to its unique micropore system, it displays an excellent performance in catalytic reactions such as 1-butene skeletal isomerization [101], methanol or ethanol dehydration [102,103] and dimethyl ether carbonylation [104,105]. However, the synthesis of FER zeolite requires the use of organic templates normally, such as cetyltrimethylammonium bromide [106], pyrrolidine [107], pyridine [108] and piperidine [109]. Undoubtedly, the use of these organic templates in synthesis increases costs and environmental concerns.

Originally, Weitkamp et al. [110] synthesized FER zeolite with good crystallinity by the addition of homonuclear FER zeolite seeds into the organotemplate-free system, but the product Si/Al ratio was low (6.6–7.8), which could not satisfy the application requirements. Later, the researchers found that FER zeolite with a high Si/Al ratio can be prepared by adding zeolite seeds with a different topological structure from FER, which was regarded as a breakthrough in the preparation of high-silica FER zeolite [73,74]. For example, Zhang et al. [73] demonstrated the successful synthesis of high-silica FER zeolite (Si/Al ratio as high as 14.5) by using RUB-37 (CDO structure) zeolite with similar secondary structural units as the zeolite seeds in the absence of an organic template.

#### 2.2.2. NES

NES zeolite owns 10-membered-ring straight channels and 12-membered-ring sinusoidal channels [111], which would be synthesized by using expensive decamethonium as an organic template [112].

**Figure 3:**
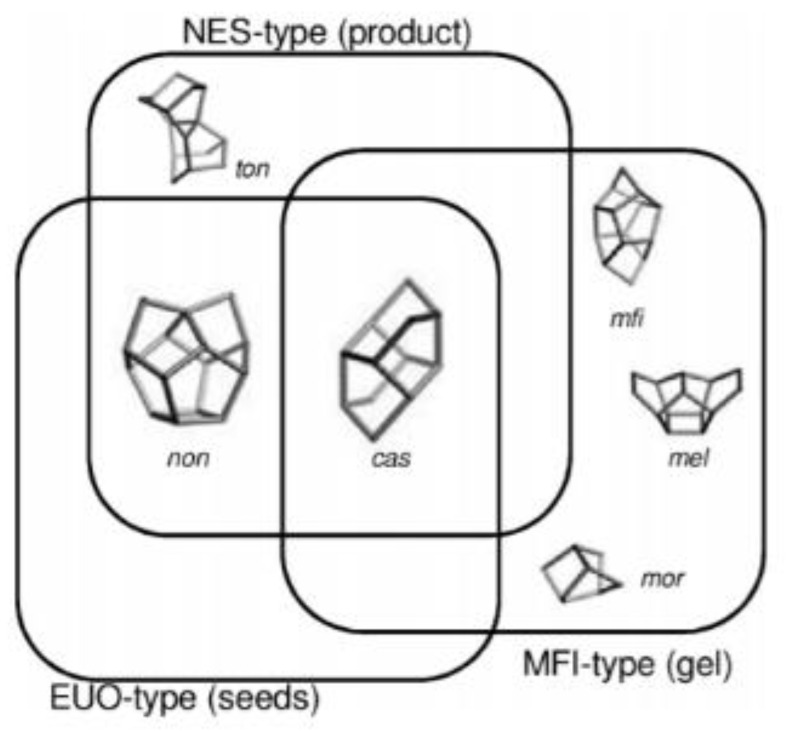
Correlation of the composite building units (CBUs) in NES-, EUO- and MFI-type zeolites. Reprinted with permission from ref. [75]. Copyright 2015, Elsevier.

Iyoki et al. [75] successfully prepared NES aluminosilicate zeolite without the addition of an organic template for the first time. The key to the successful synthesis was the use of EUO zeolite seeds with the partly same composite building units as the targeted NES zeolite product, as shown in Figure 3. In addition, the targeted NES zeolite product was affected by the initial gel composition (SiO_2_/Al_2_O_3_ and Na_2_O/SiO_2_) during the synthesis and pure NES zeolite could only be obtained in a narrow phase diagram. The micropore characteristics of the as-synthesized NES zeolite products were similar to those synthesized from conventional organic templates.

## 3. Seed-Directed Synthesis of Si-Rich Zeolites Assisted with Ethanol

### 3.1. Synthesis of All Silica Zeolite

**Figure 4:**
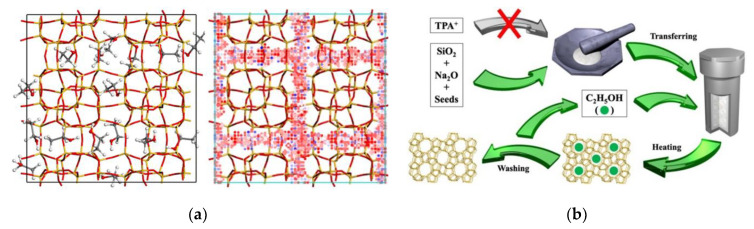
(**a**) The position of ethanol adsorbed in the micropore of silicalite-1 zeolite and the corresponding potential energy of isodensity surface; (**b**) schematic representation for synthesizing pure silica zeolites. Blue represents low potential energy. Reprinted with permission from ref. [113]. Copyright 2019, Wiley.

In fact, the zeolite products of the above-described synthesis are always Al-rich. Currently, attempts to synthesize all silica zeolites have been unsuccessful because the inorganic cations could not fill the near-neutral framework structure of all silica zeolites. In 2019, Wu et al. [113] reported that the all silica zeolites could be successfully synthesized from the combined strategy of seed directing and ethanol filling (Figure 4). To confirm the occurrence of ethanol filling in zeolite micropores, a theoretical simulation was carried out by choosing all silica ZSM-5 as a model zeolite and ethanol as a filler. The simulation results showed that there were sixteen ethanol molecules fitted per unit cell and their interaction energy (−388.1 kcal·mol^−1^) was higher than that of the conventional organic template TPABr (−405.2 kcal·mol^−1^), which proved that ethanol was a pore filler rather than a structure-directing agent. In addition, when a small amount of water was introduced into the simulated system, the interaction energy of ethanol was greatly improved, which was attributed to the presence of water destroying the hydrogen bonds among the ethanol molecules. Thus, the addition of excess water should be avoided in the synthesis.

Guided by theoretical calculations, all four silica zeolites including MFI, MTT, TON and *MRE were synthesized successfully by the above strategy [113]. The ethanol in the micropores of these all silica zeolites could be washed out fully at room temperature, thereby avoiding the calcination process, which was not only environmentally friendly, but also reduced energy consumption. As a typical example, the synthesis of all silica ZSM-5 zeolite was studied in detail. It was found that a product with high quality could only be obtained under specific synthesis conditions. Very interestingly, the ethanol in the synthetic system would be reused in the next run. In addition, the obtained zeolite product could be used as a seed again to prepare all silica ZSM-5 zeolite in the next run. More importantly, a variety of characterization methods, such as XRD, MAS NMR, TG, etc., were used for confirming that the ethanol played a role in pore filling.

Compared with the conventional synthesis, this novel strategy for synthesizing all silica zeolites had clear advantages, as follows: the avoidance of the use of costly and toxic organic templates, high product yield, and simple preparation steps, which were particularly significant for the industrial production of all silica zeolites.

More recently, Zhu et al. [114] also successfully synthesized all silica SOD, MTN and NON zeolites with six-membered ring structures from a combined strategy of ethanol filling and zeolite seeding, which further expanded the types of all silica zeolites synthesized by this route.

### 3.2. Synthesis of High-Silica Zeolite

Based on the above synthesis principles, Luan et al. [115] reported the successful synthesis of a series of high silica zeolites with MFI, MTT, TON and *MRE structures. The key to successful synthesis was the coordination of the starting aluminum species with the silica species. In the absence of organic templates, the high-silica ZSM-5 zeolite with Si/Al ratios ranging from 38 to 240 was successfully achieved from a combined strategy of both zeolite seeding and alcohol filling using four coordinated aluminum species in the starting aluminosilicate solid. Through ^29^Si MAS NMR characterization of the products in the crystallization process, it was found that the rearrangement and condensation of the silicate species only occurred during zeolite crystallization. Catalytic tests for the methanol-to-propylene (MTP) reaction displayed that high-silica ZSM-5 zeolite exhibited similar propylene selectivity and catalyst lifetimes to those of conventional products. In addition, ferrosilicate and borosilicate ZSM-5 zeolites could also be synthesized by the above synthetic method successfully, which was the first time heteroatom zeolite was synthesized without the addition of organic templates.

## 4. Utilization of Seed-Directed Synthesis

The seed-directed synthesis of zeolites greatly reduces the product cost and eliminates environmental pollution, which is a green route for zeolite synthesis. In recent years, as a result of the development of this route, more utilization of seed-directed synthesis has been reported, such as enhancing the mass transfer and preparing shaped zeolite catalysts.

### 4.1. Enhancing the Mass Transfer

It is well known that the micropore diffusion limitation of zeolite affects its performance in practical applications. In recent years, mesoporous zeolites have received extensive attention due to their excellent diffusion properties. Among them, hollow zeolites, a class of mesoporous zeolites, have been reported such as hollow TS-1 [116], hollow ZSM-5 [117] and hollow Beta [118]. However, the preparation of hollow zeolites usually requires post-treatment processes with an organic base, which was not only complicated, but also expensive. Okubo et al. [118] reported a one-pot synthesis of hollow Beta zeolite by adding CIT-6 zeolite with the same structure of Beta as zeolite seeds under an organotemplate-free condition. The key to success is that the seed-assisted synthesis of the hollow zeolite is the surface structure and dissolution rates of CIT-6 zeolite. Investigation suggested that CIT-6 zeolite seeds were not dissolved before the starting of crystal growth and intra-crystalline voids were formed by the dissolution of the seed crystals after the starting of crystal growth. This new route offered the possibility of the seed-directed synthesis of hollow zeolites without the addition of any organic templates or complicated post-processing procedures, which provided a novel strategy for the simple synthesis of other hollow zeolites.

In addition to the above-mentioned synthesis of hollow zeolites, conventional mesoporous zeolites can also greatly improve mass transfer, but their preparation often requires the use of a costly organic template or complex post-treatment. Tang et al. [119] proposed an organotemplate-free, seed-directed synthesis of ZSM-5 zeolite with abundant intracrystal mesopores (designated as Meso-Z5). The key to success is the addition of KF and aging time in the synthesis. As a result, the as-synthesized Meso-Z5 zeolite had abundant intracrystal mesopores and perfect mesopore structure, which exhibited an excellent catalytic performance in the isomerization of xylene. This facile method for preparing zeolites with abundant mesopore structures brings a new route for the development of mesoporous zeolites efficiently.

**Figure 5:**
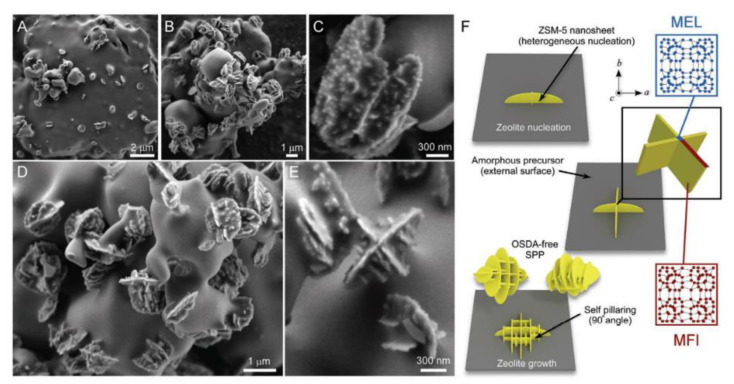
FE-SEM images of solids at different time with (**A**)1 d, (**B**) 2 d and (**C**) 3 d; (**D**,**E**) higher-magnification images of solids heated at 2 d; (**F**) schematic representation of heterogeneous nucleation and growth of SPP zeolites on amorphous interfaces. Reprinted with permission from ref. [120]. Copyright 2021, Wiley.

Moreover, the control of zeolite morphology can also improve its diffusion performance. The synthesis of zeolite nanosheets, as an effective way to improve the diffusion performance, has attracted extensive attention in recent years. In general, its preparation relies on organic templates, which greatly limits its commercial application. Recently, Jain et al. [120] first demonstrated a method for the direct synthesis of pentasil zeolite nanosheets with a “house of cards” morphology without the use of organic templates (Figure 5). It is shown that the spontaneous formation of self-pillared pentasil zeolites can be induced by using either MEL or MFI zeolite as seeds, which shows the advantages of a high-yield and high-acid site concentration compared with the conventional ZSM-5 zeolite. As a result, the self-pillared pentasil zeolites nanosheets had excellent mass transfer properties, thus exhibiting a better catalytic performance in methanol-to-hydrocarbon (MTH) and Friedel–Crafts alkylation reactions. Such a simple and efficient seed-directed synthesis method provides a new idea to produce industrial self-pillared pentasil zeolites nanosheets.

### 4.2. Synthesis of Shaped Zeolite Catalysts

In the industrial application, zeolites require the shaped form such as extrudates and pellets, which can meet the practical requirement for mechanical strength. However, the addition of the binder will not only dilute the active components, but also block the micropores of the zeolite [121]. Thus, it is of great significance to convert the binder into a corresponding zeolite product, thereby preparing binder-free zeolite catalysts. In recent years, despite some progress which has been made in the synthesis of this aspect, the synthesis of shaped zeolite catalysts is still complex and not green [122,123].

**Figure 6:**
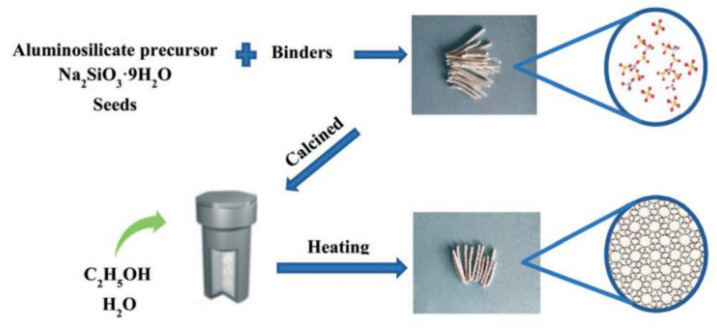
Schematic representation of the one-pot preparation of the fully crystalline-shaped zeolites catalysts. Reprinted with permission from ref. [124]. Copyright 2021, The Royal Society of Chemistry.

Wu et al. [124] reported a method in which the starting material was first shaped and then the shaped zeolite catalysts (MFI, MTT and TON) were successfully obtained from a combined strategy of zeolite seeding and ethanol filling (Figure 6). The fully crystalline-shaped ZSM-5 zeolite with an Si/Al ratio of 151 was characterized in detail and the results showed that the product obtained by this green method had many characteristics, such as high crystallinity, a uniform crystal size, good mechanical strength, a high surface area and a unique core–shell structure. In the MTP reaction, this fully crystalline-shaped ZSM-5 zeolite exhibited an extremely long reaction lifetime. This work had obvious advantages as follows: the avoidance of the use of toxic and expensive organic templates, being a simple procedure, being a calcination-free process, the reduction of waste emissions, an extremely high product yield and an excellent catalytic performance, which provides a new idea for the large-scale production of industrial-shaped zeolite catalysts.

## 5. Conclusions and Prospects

In this review, the synthesis of Al-rich zeolites with the assistance of homonuclear and heteronuclear seeds, the synthesis of Si-rich zeolites from a combined strategy of zeolite seeding and ethanol filling and the research on the utility of the seed-directed method are reviewed. The summary of zeolites prepared from the seed-directed route with optimal synthetic conditions and their key properties discussed in the review is shown in Table 2. The seed-directed synthesis of zeolites is green, simple and cheap, compared with the conventional synthesis of zeolite in the presence of organic templates. In addition, the seed-directed synthesis of zeolites, as one of the important progresses in the zeolite field, can promote the development of this field both from the perspective of fundamental research and industrial applications. Currently, we believed that more zeolites are prepared in this way, using this green route.

In the past decade, although the seed-directed synthesis of zeolite has made great progress, it still has some shortcomings, such as a low product yield and a narrow phase diagram. In addition, the types of zeolites that can be industrially produced by this route are very limited. Up to now, only Beta and ZSM-22 zeolites have been successfully industrialized in the reported literature [125]. It is believed that more zeolites could be industrially prepared by the seed-directed route in the near future. Moreover, because the directing ability of zeolite seeds is much lower than that of an organic template, the scale-up of the above zeolites is much more difficult than that of conventional production in the presence of organic templates. One of the typical production parameters is the stirring rate, which should be carefully adjusted in the production process. In addition, due to the more inorganic cations in the micropore of zeolites, more ion-exchange would be necessary, which also increases the difficulty of production.

Considering that the large-scale production of zeolites can produce huge social and economic benefits, it is urgent to develop a new green route for synthesizing zeolites such as the seed-directed route. In conclusion, designed synthesis for zeolites should be done continually in the future.

## Figures and Tables

**Table 1:** List of zeolites prepared from the seed-directed route and their characteristics.

Run	Zeolite	IZA Code	Dimension	Pore Size	References
1	Beta	*BEA	3	0.66 nm × 0.77 nm	[36,37,38]
2	ZSM-23	MTT	1	0.56 nm × 0.65 nm	[39]
3	SSZ-13	CHA	3	0.45 nm × 0.56 nm	[40,41,42]
4	MCM-22	MWW	2	0.38 nm × 0.38 nm	[43]
5	MCM-49	MWW	2	0.40 nm × 0.59 nm	[44]
6	SUZ-4	SZR	3	0.70 nm × 0.71 nm × 0.71 nm	[45]
7	MCM-68	MSE	3	0.71 nm × 0.71 nm × 1.82 nm	[46]
8	ZK-5	KFI	3	0.40 nm × 0.59 nm	[47,48]
9	RUB-13	RTH	2	0.70 nm × 0.71 nm × 0.71 nm	[49,50]
10	ZSM-12	MTW	1	0.71 nm × 0.71 nm × 1.82 nm	[51,52,53]
11	ZSM-22	TON	1	0.46 nm × 0.52 nm	[54]
12	ZSM-5	MFI	3	0.64 nm × 0.68 nm	[55,56,57,58]
13	ZSM-11	MEL	3	0.52 nm × 0.58 nm × 0.52 nm	[58]
14	ECR-18	PAU	3	0.39 nm × 0.39 nm	[58]
15	Omega	MAZ	1	0.41 nm × 0.38 nm	[59,60,61]
16	VPI-8	VET	1	0.56 nm × 0.25 nm	[62]
17	ITQ-47	BOG	3	0.7 nm × 0.55 nm × 0.58 nm	[63]
18	ITQ-58	-	3	0.4 nm × 0.33 nm 0.59 nm × 0.25 nm	[64]
19	EU-1	EUO	1	0.41 nm × 0.54 nm	[65]
20	SSZ-35	STF	1	0.54 nm × 0.57 nm	[66]
21	ECR-1	EON	2	0.67 nm × 0.68 nm 0.34 nm × 0.49 nm 0.29 nm × 0.29 nm	[67]
22	ZSM-34	ERI/OFF	3	0.36 nm × 0.51 nm 0.67 nm × 0.68 nm 0.36 nm × 0.49 nm	[68]
23	Heulandite	HEU	2	0.31 nm × 0.75 nm 0.36 nm × 0.46 nm 0.28 nm × 0.47 nm	[69]
24	ZK-20	LEV	2	0.36 nm × 0.48 nm	[70,71]
25	EMC-2	EMT	3	0.73 nm × 0.65 nm × 0.75 nm	[72]
26	FER	FER	2	0.42 nm × 0.54 nm 0.35 nm × 0.48 nm	[73,74]
27	NU-87	NES	2	0.48 nm × 0.57 nm	[75]

**Table 2:** List of zeolites prepared from the seed-directed route with optimal synthetic conditions and their key properties.

Run	Zeolite	IZA Code	The Best Synthesis Conditions					Characteristics			References
			Tem. (°C)	Time (h)	Gel Composition	Preparation Steps	Initial Si/Al	BET (m^2^/g)	Crystal Diameters	Phase Purity	
1	Beta	*BEA	140	18.5	NaAlO_2_-NaOH- fumed silica- H_2_O-Beta seeds	Homonuclear seeds	20	632	100–160 nm	100%	[36,37]
2	Beta	*BEA	140	96	silica sol-Al(OH)_3_- NaOH-H_2_O-Beta seeds	Homonuclear seeds	5.5–10	-	1–2 μm	100%	[38]
3	ZSM-23	MTT	170	5	NaOH-Al_2_(SO_4_)_3_- H_2_O-silica sol- ZSM-23 seeds	Homonuclear seeds	60	117	100 nm	100%	[39]
4	SSZ-13	CHA	170	24	NaAlO_2_-NaOH- KOH/CsOH- fumed silica- Na_2_B_4_O_7_-SSZ-13 seeds	Homonuclear seeds	10	574	0.07–0.15 μm	100%	[40]
5	SSZ-13	CHA	160	48	NaOH-aluminum isopropoxide-H_2_O- fumed silica-SSZ-13 seeds	Homonuclear seeds	19.2	623	3–6 μm	100%	[41]
6	MCM-22	MWW	160	96	fumed silica- NaAlO_2_-NaOH- MCM-22 seeds	Homonuclear seeds	200	-	-	100%	[43]
7	MCM-49	MWW	140	32	silica sol- NaAlO_2_-MCM-49 seeds	Ultraphonic aging, Homonuclear seeds	20	245	150 nm	100%	[44]
8	ZSM-5	MFI	180	48	Na_2_SiO_3_·9H_2_O- silica gel-ethanol- silicalite-1 seeds	Homonuclear seeds	Pure silica	385	1 μm	100%	[113]
9	ZSM-5	MFI	180	48	Na_2_SiO_3_·9H_2_O- aluminosilicate precursor-ethanol- ZSM-5 seeds	Homonuclear seeds	80	382	1 μm	100%	[114]
10	Intracrystal mesoporous ZSM-5	MFI	180	6	silica sol-Al_2_(SO_4_)_3_- Na_2_O-KF-H_2_O- silicalite-1 seeds	Homonuclear seeds	34	481	4 mm	100%	[119]
11	SPP zeolite	MFI	150	72	NaOH-silica sol- NaAlO_2_-H_2_O- silicalite-1 seeds	Homonuclear seeds	9	499	30 nm	100%	[120]
12	Shaped ZSM-5	MFI	180	96	Na_2_O- aluminosilicate precursor- H_2_O-ZSM-5 seeds	Homonuclear seeds	80	335	1–2 μm	100%	[124]
13	SOD	SOD	140	96	Na_2_SiO_3_·9H_2_O-silica gel-ethanol-SOD seeds	Homonuclear seeds	Pure silica	-	1 μm	100%	[115]
14	Hollow Beta	*BEA	140	46	Na_2_O-Al_2_O_3_- fumed silica-H_2_O-CIT-6 seeds	Homonuclear seeds	50	614	500 nm	100%	[118]
15	FER	FER	150	72	NaAlO_2_-NaOH- H_2_O-fumed silica- RUB-37 seeds	Heteronuclear seeds	15.9	370	2–4 μm	100%	[73]
16	FER	FER	165	16	NaAlO_2_-NaOH- H_2_O-silica sol- MCM-49 seeds	Heteronuclear seeds	60	365	2–5 μm	100%	[74]
17	NU-87	NES	160	84	NaAlO_2_-NaOH- H_2_O-fumed silica- EU-1 seeds	Heteronuclear seeds	120	420	500 nm	100%	[75]

## Data Availability

Not applicable.
